# Development of a novel clinical scoring system for on-farm diagnosis of bovine respiratory disease in pre-weaned dairy calves

**DOI:** 10.7717/peerj.238

**Published:** 2014-01-02

**Authors:** William J. Love, Terry W. Lehenbauer, Philip H. Kass, Alison L. Van Eenennaam, Sharif S. Aly

**Affiliations:** 1 Veterinary Medicine Teaching and Research Center, School of Veterinary Medicine, University of California - Davis, Tulare, CA, USA; 2 Department of Population Health and Reproduction, School of Veterinary Medicine, University of California - Davis, Davis, CA, USA; 3 Department of Animal Science, University of California - Davis, Davis, CA, USA

**Keywords:** Bovine respiratory disease, Dairy calves, Clinical scoring system, BRD

## Abstract

Several clinical scoring systems for diagnosis of bovine respiratory disease (BRD) in calves have been proposed. However, such systems were based on subjective judgment, rather than statistical methods, to weight scores. Data from a pair-matched case-control study on a California calf raising facility was used to develop three novel scoring systems to diagnose BRD in preweaned dairy calves. Disease status was assigned using both clinical signs and diagnostic test results for BRD-associated pathogens. Regression coefficients were used to weight score values. The systems presented use nasal and ocular discharge, rectal temperature, ear and head carriage, coughing, and respiratory quality as predictors. The systems developed in this research utilize fewer severity categories of clinical signs, require less calf handling, and had excellent agreement (Kappa > 0.8) when compared to an earlier scoring system. The first scoring system dichotomized all clinical predictors but required inducing a cough. The second scoring system removed induced cough as a clinical abnormality but required distinguishing between three levels of nasal discharge severity. The third system removed induced cough and forced a dichotomized variable for nasal discharge. The first system presented in this study used the following predictors and assigned values: coughing (induced or spontaneous coughing, 2 points), nasal discharge (any discharge, 3 points), ocular discharge (any discharge, 2 points), ear and head carriage (ear droop or head tilt, 5 points), fever (≥39.2°C or 102.5°F, 2 points), and respiratory quality (abnormal respiration, 2 points). Calves were categorized “BRD positive” if their total score was ≥4. This system correctly classified 95.4% cases and 88.6% controls. The second presented system categorized the predictors and assigned weights as follows: coughing (spontaneous only, 2 points), mild nasal discharge (unilateral, serous, or watery discharge, 3 points), moderate to severe nasal discharge (bilateral, cloudy, mucoid, mucopurlent, or copious discharge, 5 points), ocular discharge (any discharge, 1 point), ear and head carriage (ear droop or head tilt, 5 points), fever (≥39.2°C, 2 points), and respiratory quality (abnormal respiration, 2 points). Calves were categorized “BRD positive” if their total score was ≥4. This system correctly classified 89.3% cases and 92.8% controls. The third presented system used the following predictors and scores: coughing (spontaneous only, 2 points), nasal discharge (any, 4 points), ocular discharge (any, 2 points), ear and head carriage (ear droop or head tilt, 5 points), fever (≥39.2°C, 2 points), and respiratory quality (abnormal respiration, 2 points). Calves were categorized “BRD positive” if their total score was ≥5. This system correctly classified 89.4% cases and 90.8% controls. Each of the proposed systems offer few levels of clinical signs and data-based weights for on-farm diagnosis of BRD in dairy calves.

## Introduction

Bovine respiratory disease (BRD) is a major source of economic loss for the cattle industry (Panciera & Confer, 2010; Sischo et al., 1990; USDA, 2008). Respiratory disease is the leading cause of death in weaned dairy heifers and the second most common cause of mortality in pre-weaned calves in cattle herds in the United States. It is estimated that BRD is responsible for the loss of more than one million animals and approximately US $700 million annually (USDA, 2007; Wittum et al., 1996). Effective control of BRD has proven difficult in the North American dairy industry, at least in part due to the complexity of disease pathogenesis and the ubiquity of BRD-associated pathogens (Gorden & Plummer, 2010).

The healthy bovine respiratory tract has several mechanisms which prevent harmful microorganisms from colonizing exposed tissues, including mucous and cilia to trap and physically remove microbes and particulates, the mucosal immune response, and the maintenance of a symbiotic population of commensal bacteria (Ackermann, Derscheid & Roth, 2010). When infected by primary respiratory pathogens, such as bovine respiratory syncytial virus (BRSV) (Brodersen, 2010), bovine viral diarrhea virus (BVDV) (Ridpath, 2010), bovine herpesvirus type 1 (Jones & Chowdhury, 2010), or Parainfluenza 3 (PI3) virus (Ellis, 2010), the host’s respiratory defenses may become impaired (Ames, 2002; Caswell & Williams, 2007). *Pasteurella multocida*, *Histophilus somni*, *Mannheimia haemolytica* (Griffin et al., 2010), and *Mycoplasma bovis* (Caswell et al., 2010), may be naturally present in small numbers in the nasal passages of healthy cattle but can be opportunistically pathogenic when the host’s defenses become impaired. Other factors including nutritional status, stress, and air quality may also play roles in impairing host defense mechanisms (Ackermann, Derscheid & Roth, 2010; Gorden & Plummer, 2010).

Respiratory disease in calves may involve the upper or lower respiratory tract (Panciera & Confer, 2010). Infections of the upper respiratory tract such as rhinitis typically present with ocular and nasal discharge. Infections of the lower tract, in contrast, may be challenging to detect earlier in the disease course. In addition, both upper and lower tract infections may vary in severity. Despite the variability of presentation, the observation of clinical signs is the most common method used to identify cattle in need of treatment for BRD. However, the specific criteria used to detect BRD are subjective and vary widely among dairies and observers, leading to deleterious effects on animal welfare and unnecessary treatments with antimicrobial drugs (Kelly & Janzen, 1986; Radostits & Done, 2007). Identification of etiologic agents associated with specific cases of BRD based on clinical observation alone is not typically possible.

Respiratory disease can be confirmed using a variety of methods. Necropsy and diagnostic testing for BRD pathogens is the gold standard test to diagnose BRD. Imaging modalities, such as thoracic ultrasound and radiography, are also available to diagnose BRD ante mortem, but rely on expensive equipment that require specialized training to use and interpret (Abutarbush et al., 2012; Curtis et al., 1986; Masseau et al., 2008). Molecular and biochemical diagnostic tests, such as PCR and culture on selective media, respectively, are available for ante mortem diagnosis of BRD, but are prohibitively expensive and cannot provide results at the point of treatment (Cooper & Brodersen, 2010). Necropsy and molecular and biochemical methods may also be used to identify etiologic agents associated with cases of BRD; however, such identification is not a routine practice unless BRD has become epidemic in a herd.

A simple, objective clinical scoring system to improve and standardize BRD identification in dairy calves without the need for expensive equipment would be a useful tool for farm workers, clinicians, and researchers. Clinical scoring systems use information that can be rapidly collected from patients to assess patient health and prognosis and have been used in a variety of human and veterinary applications (Champion et al., 1981; Champion et al., 1989; Sullivan, Massaro & D’Agostino, 2004; Tollner, 1982). Scoring systems assign values to clinical signs, which are used to determine a total score. The patient’s total score, in turn, should correspond to their risk or likelihood of disease. Objective methods should be used to weight scores using clinical data to ensure that similar scores represent similar risks and to optimize score performance. Clinical signs that are difficult to measure with adequate precision or that require expensive or time-consuming methods to measure should not be included (Sullivan, Massaro & D’Agostino, 2004).

Clinical scoring systems for BRD are not novel, and at least three scoring systems have previously been described to diagnose BRD in cattle. The first score published was developed by Thomas et al., as a research tool to quantitatively classify the severity of BRD in calves experimentally inoculated with BRSV or BVDV (Thomas et al., 1977). More recently, a scoring system was developed by veterinarians at the University of Wisconsin at Madison (McGuirk, 2008) and is based on five clinical signs to identify calves that should be treated for BRD. A third system known as DART (Depression, Appetite, Respiration and Temperature) was developed to identify beef cattle for BRD treatment in feedlots (Panciera & Confer, 2010).

The score described by Thomas et al. (1977) is ill-suited for field work because it uses 17 predictors, hematologic data, and requires observations specific to the pre-inoculation described in their study. The DART system does not appear in peer-reviewed literature and is difficult to standardize between locations because the clinical sign weights and decision points are not defined. The WI score is the most suitable of the three cited scores with published score weights and a decision rule. However, the WI score subdivides each of its clinical signs into 4 levels, which may have ambiguous overlap to inexperienced individuals making it difficult to appropriately classify clinical signs in calves. Until recently, information regarding these systems’ diagnostic performance in the field have not been published. A single published study has estimated the sensitivity and specificity of the WI scoring system as 55.4% and 58.0%, respectively (Buczinski et al., 2013). There is also no evidence that any of these BRD scores used quantitative methods to assign weights to clinical signs.

The objective of this study was to develop a simple scoring system with objectively assigned score weights for on-farm diagnosis of BRD in pre-weaned dairy calves. The scoring system developed in this study will be validated and used as part of a risk assessment tool under development. The risk assessment tool will be used to identify farm-specific management practices associated with BRD. A similar approach is being used to control and prevent the transmission of Johne’s disease in dairy herds (Berghaus et al., 2005).

## Materials & Methods

Data used in this study were from a separate study performed to identify single nucleotide polymorphisms (SNPs) associated with BRD susceptibility. The original genetic marker research was a case-control study in which clinically ill cases were pair-matched to clinically healthy controls. The study was approved by the University of California, Davis Institutional Animal Care and Use Committee (protocol number 16431, approval date March 30, 2011).

### Study population and sampling

Study calves were enrolled on a calf raising facility in California’s San Joaquin Valley that housed between 60,000 and 80,000 calves and specialized in raising dairy bull, steer, and heifer calves. All bull and steer calves raised on the facility were purchased from dairies and raised for beef. Heifer calves were raised on contract as replacement stock for client dairies. Calves were typically 1–2 days old at arrival and were segregated according to size and weight. Facility personnel also collected serum from all calves upon arrival to measure serum total protein via refractometer to identify calves at risk of failure of passive transfer (FPT). Calves were individually housed in clean, sanitized, 3 feet by 6 feet wooden hutches, arranged in rows of 480 hutches. Calves were able to have nose-to-nose contact with adjacent calves only. Calves were typically moved from hutches to group pens at 70 days of age, but the age varied based on the needs of the facility.

Calves were vaccinated with a modified-live intranasal vaccine against BHV-1 and PI3 upon arrival, at approximately one day of age. A 5-way modified-live parenteral vaccine against BHV-1, BVDV types 1 & 2, PI3, and BRSV was administered at 8 days of age. A *Moraxella bovis* bacterin was administered at approximately 65 days of age, prior to the calves being moved to group pens.

Calves were enrolled between July 2011 and January 2012. Calves older than 22 days were enrolled to allow at least 14 days after vaccination to avoid false positive tests caused by detection of vaccine virus (Timsit et al., 2009). Similarly, calves treated with antibiotics were not eligible to be enrolled for 10 days after final treatment due to concerns that treatment could affect bacterial culture results.

On any study day, two or three veterinarians and trained staff researchers visually evaluated calves for possible signs of BRD, including abnormal respiration, mentation, and head and neck carriage. Approximately 25 rows (12,000 calves) of calves were eligible for enrollment on any day during the study, however, only 4–6 rows per day could be evaluated due to time limitations. Evaluation occurred between 6 and 9 AM on study days. Calves were typically awake during this time in anticipation of the morning feeding allowing for assessment of mentation.

Calves suspected to have BRD were scored using the WI BRD clinical scoring system summarized in Table 1 (McGuirk, 2008). The evaluation included a member of the research team entering the hutch to measure the calf’s body temperature using a rectal thermometer and manipulate the calf’s larynx to determine if a cough could be induced. Information on nasal discharge, ocular discharge, ear and head carriage, rectal temperature, and the frequency of induced and spontaneous coughing were recorded. Calves suspected to have BRD and that had a WI BRD score of 5 or greater were classified as clinically positive for BRD and enrolled. Calves suspected to have BRD were not enrolled if they had WI scores of 4 or less. For each clinically positive BRD calf, a calf in an adjacent hutch with a WI score of 4 or less was enrolled and pair-matched to the clinically ill calf. If a suitable calf could not be found in an adjacent hutch, the nearest healthy calf in the same row with a WI score of 4 or less was enrolled and pair-matched to the clinically ill calf instead.

**Table 1:** Summary of the scoring system^a^ for bovine respiratory disease (BRD) designed by researchers at the University of Wisconsin at Madison^b^. Clinical signs scored “0” are considered to be clinically normal.

	Score			
	0	1	2	3
Cough	None	Single induced	Multiple induced	Multiple spontaneous
			Few/occasional spontaneous	
Nasal discharge	None	Small amount of unilateral cloudy discharge	Bilateral, cloudy, or excessive mucus discharge	Copious bilateral mucopurulent discharge
Ocular discharge	None	Small amount of ocular discharge	Moderate amount of bilateral discharge	Heavy ocular discharge
Ear & Head carriage	Normal carriage	ear flick or head shake	slight unilateral droop	Head tilt or bilateral droop
Rectal temperature (F)	≤100.9	101.0–101.9	102.0–102.9	≥103.0

**Notes.**

a The total WI score each calf was assigned the sum of the nasal discharge, rectal temperature, cough scores and the greater one of the two scores from the ocular discharge and head/ear carriage.

b http://www.vetmed.wisc.edu/dms/fapm/fapmtools/8calf/calf_health_scoring_chart.pdf.

Clinical signs beyond those used in the WI score were also observed and recorded. Calves were noted to be depressed based on observed clinical attitude and behavior. Diarrhea was noted if the calf’s fresh feces had a loose or watery consistency. Calves with very poor body condition were noted to be emaciated. Tachypnea was noted if the calf’s respiratory rate was noticeably elevated compared to other nearby calves. Dyspnea was noted if the calf had a noticeable abdominal component to their respiration.

### Biologic sample collection

After all clinically positive BRD calves and clinically negative BRD calves were scored, the research team revisited each newly enrolled calf to collect samples for diagnostic testing. Swabs for bacterial cultures and viral detection via PCR were collected from the calves’ upper respiratory tracts (Fulton & Confer, 2012). An unguarded sterile polyester swab was placed through a single naris and into the nasopharynx of each calf, repeatedly passed over the nasal mucosa, and the tip stored in a vial containing a viral transport medium. A second sample was collected from the pharyngeal recess by passing a guarded polyester swab through a naris and along the ventral nasal meatus. This swab tip was stored in the same vial of viral transport media. The vial was sealed and stored on wet ice until daily sampling was completed, then stored at −80°C. The nasopharyngeal and pharyngeal recess swabs were submitted to the Davis branch of the California Animal Health and Food Safety Laboratory (CAHFS) for viral respiratory pathogen testing. A real-time quantitative PCR (qPCR) panel was performed to detect BRD-associated viruses. The panel included qPCR assays for bovine herpesvirus-1 (BHV-1) (Brower et al., 2008), bovine respiratory syncytial virus (BRSV) (Boxus, Letellier & Kerkhofs, 2005), bovine viral diarrhea virus (BVDV) (Mahlum et al., 2002) and bovine coronavirus (BCoV) (Dr. K. Kurth, Wisconsin Veterinary Diagnostic Laboratory, WI Madison, unpublished data).

A second guarded polyester swab was similarly collected from the pharyngeal recess, stored in Brucella broth + 10% glycerol and submitted to the Tulare branch of CAHFS for aerobic and mycoplasma bacterial cultures. Aerobic bacterial cultures were performed by plating broth onto blood and chocolate agar plates at 37°C for 48 h. Individual colonies were re-plated and cultured for identification. Organisms were identified based on colony morphology and confirmed by biochemical characteristics. Samples for *Mycoplasma* spp. were cultured in enrichment broth for 48 h, then plated on modified Hayflick agar and incubated in CO_2_ for up to 7 days. Colonies of *Mycoplasma* spp. were identified by colony morphology and confirmed with digitonin (Thurmond, Holmberg & Luiz, 1989) and Diene’s stain (Dienes & Weinberger, 1951).

The media used for sample collection and storage was supplied by the University of California Davis’ Veterinary Medical School’s Biological Media Service (BMS). The viral transport media (BMS product #5404) contained minimal essential medium, sodium bicarbonate, HEPES buffer, Gentamycin, Amphotericin B, and water. The Brucella broth (BMS product #5571) contained pancreatic digest of casein, peptic digest of animal tissue, dextrose, yeast extract, sodium chloride, sodium bisulfite, and 10% glycerol.

Whole blood and serum were also collected from each enrolled calf. The samples were genotyped with the High-Density Bovine BeadChip array product. Bovine HD (Illumina Inc., San Diego, CA) as part of a whole genome association study to identify loci associated with susceptibility to BRD. Results of the genetic analyses are the subject of a different report.

### Case definition

**Figure 1:**
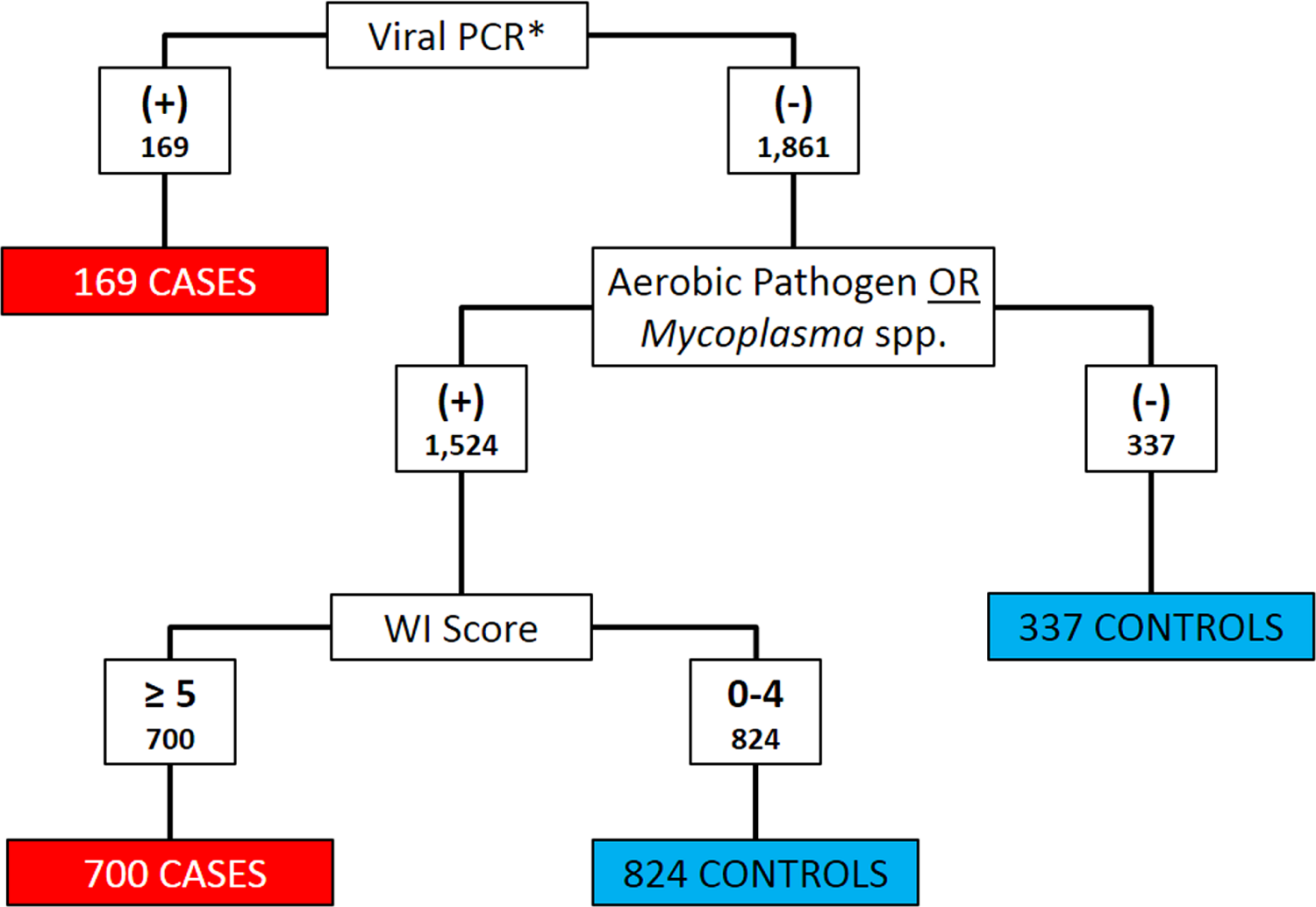
Flowchart depicting the decision rules used to assign 2030 Holstein calves as BRD cases or healthy controls. BRD case status determined using qPCR for bovine respiratory syncytial Virus (BRSV), bovine viral diarrhea virus (BVDV) and bovine herpesvirus-1 (BHV-1), aerobic pathogen culture results, *Mycoplasma* spp. culture results, and the University of Wisconsin at Madison clinical scoring system (http://www.vetmed.wisc.edu/dms/fapm/fapmtools/8calf/calf_health_scoring_chart.pdf). Organisms considered to be aerobic pathogens included *Pasteurella multocida*, *Mannheimia haemolytica*, *Bibersteinia trehalosi* and *Histophilus somni*. *All Viral qPCR positive results were positive for BRSV. No samples were reported to be qPCR positive for BHV-1 or BVDV.

Clinical signs and diagnostic test results were both used to classify calves as BRD cases or controls. *Histophilus somni*, *Pasteurella multocida*, *Bibersteinia trehalosi* and *Mannheimia haemolytica* were considered as aerobic pathogens when categorizing cases and controls (Griffin et al., 2010). Calves that met any of the following three criteria were classified as cases: (1) positive for BRSV, BHV-1, or BVDV on PCR; (2) any aerobic pathogen detected on culture and WI Score ≥5; or (3) any *Mycoplasma* spp. detected on culture and WI Score ≥5. All other calves were classified as controls. Figure 1 depicts the algorithm for the classification of BRD cases and controls. Bovine coronavirus was not included as a criterion for case definition because current PCR assays could not differentiate between enteric and respiratory BCoV subtypes (Saif, 2010).

### Statistical analysis

Conditional logistic regression (CLR) was used to analyze the data and estimate the exposure odds ratios relating case status to clinical signs while accounting for the pair-matching in the original study design (Breslow & Day, 1980). Pair identifiers from the original study were used to define pairs. Due to matching, the distribution of date of birth, source farm, and age were similar in cases and controls due to the association between these factors and calf location. Similarly, the distribution of the ambient temperatures at the time of sampling was comparable between cases and controls due to matching on time. Hence, the effects of these correlated factors were conditioned out of the analysis.

The CLR model used is summarized in Eq. (1) (Breslow & Day, 1980). In this notation, *P* represents the set of all clinical signs included in the equation, *p* represents specific levels of clinical signs included in the model, *j* represents a case-control pair matched on hutch location and time, and *X*_1*j*_ and *X*_0*j*_ are *P* × 1 vectors that, respectively, represent the case’s and control’s observed clinical signs from the *j*th pair. The resulting odds ratios compare the odds of the presence of a clinical sign severity level in a case to the odds of the presence of the clinical sign severity in a pair-matched control calf.

(1) \begin{eqnarray*} \displaystyle \ln \left({O R}_{{X}_{1}\hspace{0.167em} \text{vs}\hspace{0.167em} {X}_{0}\vert j}\right)=\sum _{p}^{P}{\beta }_{p}\left({X}_{1 j p}-{X}_{0 j p}\right)&&\displaystyle \end{eqnarray*}

Due to the revision of case definitions based on diagnostic test data available after sampling, some original case-control pairs contained only 2 controls or only 2 cases rendering them invalid for CLR analysis.

A categorical form of each of the five WI score clinical signs was forced into all models. In the original study, rectal temperature (*X*_rectal_temp_) was recorded as a continuous variable in Fahrenheit to the nearest tenth degree. This variable was dichotomized and recorded into a new predictor variable, which was coded 0 if *X*_rectal_temp_ < 39.2°C (102.5°F) and 1 if *X*_rectal_temp_ ≥ 39.2°C (102.5°F). The threshold was selected based on the reported physiologic upper limit of the normal rectal temperature range of calves, 39.2°C (Andersson & Jonasson, 1993). Therefore, rectal temperatures in excess of 39.2°C may be considered febrile and consistent with an inflammatory response to BRD. Rectal temperatures below the reported normal physiologic lower limit, 38.1°C, were not considered to be abnormal for the purposes of this study. The remaining WI score clinical signs (cough, nasal discharge, ocular discharge, head/ear carriage) were categorical predictors, and recoded into sets of dichotomous dummy variables (Breslow & Day, 1980).

Additional clinical predictors were recorded as present or absent, and were coded 1 and 0, respectively. A new variable was created to indicate abnormal respiration and was coded as 1 for dyspneic or tachypneic calves and 0 for eupneic calves.

Different strategies were used to re-categorize the levels of nasal discharge, ocular discharge, ear and head carriage and cough with the goal of simplifying the scoring. The strata that indicated a normal clinical presentation of each sign was used as the referent. Levels of clinical predictors with ORs that were non-significant or with estimated ORs that were similar to adjacent strata were merged into a single stratum. Levels of clinical predictors that converged poorly due to sparse sample size within strata were also collapsed into adjacent strata to improve convergence. Other clinical signs were added to the model using a forward selection method and model fit compared using likelihood ratio tests (LRT). The LRT method evaluates the change in deviance (ΔG^2^) caused by the addition of a term to a model (Kleinbaum & Kleinbaum, 1998), hence, was used to compare nested models. The Akaike Information Criterion (AIC) was used to compare non-nested models with models with lower AIC values preferred over models with higher AIC values (Akaike, 1974). All comparisons were considered significant at an alpha less than 0.05. The final selected models were used as the source to generate the score weights for the clinical signs.

### Scoring system development

A score weight (*S_p_*) was assigned to each severity level of clinical sign included in the final model. The magnitude of *S_p_* was defined as the value of the corresponding regression coefficient β on the natural logarithm scale rounded to the nearest integer. The total score for each calf (*S*_total,*i*_; where *i* denotes a unique calf), was calculated as the sum of the *S_p_* values corresponding to the calf’s observed abnormal clinical signs. This approach is adapted from a previous score development method (Segev et al., 2008).

(2) \begin{eqnarray*} \displaystyle {S}_{p}=\left[{\beta }_{p}\right]&&\displaystyle \end{eqnarray*}

(3) \begin{eqnarray*} \displaystyle {S}_{\mathrm{total},i}=\sum _{p}^{P}{{X}_{i p}S}_{p}&&\displaystyle \end{eqnarray*}

The decision rule to interpret the score results was to classify a calf as test positive if the total score was greater than a critical point, *S_cp_*, and test negative otherwise.

\begin{eqnarray*} {S}_{\mathrm{total},i}~i s~\left\{\begin{array}{@{}ll@{}} \displaystyle {\geq }{S}_{c p},&\displaystyle \text{then test positive for BRD}\\ \displaystyle {\lt }{S}_{c p},&\displaystyle \text{then test negative for BRD} \end{array}\right. \end{eqnarray*}

A positive test result was considered concordant with cases and a negative test result concordant with controls. Test performance was evaluated at all possible cut-point values (*S_cp_*) using receiver operating characteristic (ROC) curve analysis. The positive likelihood ratio for each cut-point was also calculated using Eq. (4).

(4) \begin{eqnarray*} \displaystyle L R+=\frac{P r\left({S}_{\mathrm{total}}\geq {S}_{c p}\vert \mathrm{BRD}+\right)}{1-P r\left({S}_{\mathrm{total}}\lt {S}_{c p}\vert \mathrm{BRD}-\right)}&&\displaystyle \end{eqnarray*}

The *S_cp_* that correctly assigned concordant results to the greatest proportion of calves in the data set was defined as the optimal cut-point (*S*_*cp*,optimal_) for that scoring system.

Cohen’s kappa coefficients were calculated as a measure of inter-rater agreement between the results of each scoring system developed and the WI system (Cohen, 1960). Kappa coefficient values less than 0.4 indicated poor agreement beyond chance, values between 0.4 and 0.75 indicated fair to good agreement beyond chance, and greater than 0.75 indicated excellent agreement beyond chance (Fleiss, Levin & Paik, 2003).

## Results

The data set included clinical findings, viral PCR, and Mycoplasma and aerobic culture results for 2,030 calves. All calves enrolled in the study were Holsteins and were between 23 and 69 days of age when sampled. Bovine respiratory syncytial virus was detected in the upper respiratory tract of 169 (8.3%) calves. Bovine coronavirus was detected in 168 (8.3%) of calves. No calves tested positive for BVDV or BHV-1 virus. Pathogenic aerobes were cultured from the pharyngeal recess swabs of 911 calves, and *Mycoplasma* spp. were cultured from the pharyngeal recesses of 1,234 calves. At least one pathogen (BRSV, Mycoplasma spp., *Histophilus somni*, *Pasteurella multocida*, *Bibersteinia trehalosi* or *Mannheimia haemolytica*) was detected in 811 (87.2%) enrolled calves. A total of 932 calves (45.9%) had a WI BRD score of 5 or greater.

Eight hundred sixty-nine calves were classified as cases and 1,161 calves were classified as controls using the algorithm in Fig. 1. Only 809 pairs of the pairs enrolled contained both one case and one control and were valid to be included in the conditional logistic regression analysis. Twenty-eight pairs contained 2 cases and 166 pairs contained 2 controls. Twenty-four calves were not assigned to a pair in the data set.

Depression was noted in 530 (61.0%) cases, and 54 (4.4%) controls and the crude pair-matched OR for depression was 171.7 (95% CI: (55.2, 534.0), *p* < 0.0005). Diarrhea was observed in 26 cases and 3 controls, however, a pair-matched OR could not be estimated since no pairs had a control with and control without diarrhea. Six cases and zero controls were emaciated. A pair-matched OR for emaciation could not be estimated since no emaciated controls were sampled. Tachypnea was noted in 455 (52.4%) cases and 51 (4.4%) controls. The crude pair-matched OR for tachypnea was 218.5 (95% CI: (54.5, 876.4), *p* < 0.0005). Dyspnea was noted in 172 (19.8%) cases and 12 (1.0%) controls. The crude pair-matched OR for dyspnea was 164 (95% CI: (23, 1171), *p* < 0.0005). Abnormal respiration was noted in 467 (53.7%) cases and 52 (4.5%) controls. The crude pair-matched OR for abnormal respiration was 224.5 (95% CI: (56.0, 900.4), *p* < 0.0005). Diarrhea and emaciation were not further considered as candidates to be included in the model since pair-matched ORs could not be estimated.

Upon arrival to the facility, 332 (38.3%) cases and 329 (28.3%) controls had serum total protein concentrations equal to or less than 5.2 g/dl, which is consistent with failure of passive transfer of maternal antibodies. The crude pair-matched OR for FPT was 1.6 (95% CI: (1.3, 2.1), *p* < 0.0005).

### Logistic regression models

#### BRD1

The first model selection process started with a model that included all levels of severity for the cough, nasal discharge, ocular discharge, and head and ear position clinical signs as described in the WI BRD score, and rectal temperature dichotomized at 39.2°C.

The simplest and best-fit model that resulted from the selection process included variables for the five WI score clinical sign variables and abnormal respiration. The variables for cough, nasal discharge, and ocular discharge were each dichotomized with a referent level for normal signs (WI BRD score = 0) and a second level for any abnormal signs (WI BRD score = 1, 2, or 3). The head and ear position was dichotomized with a referent level that included normal head position (WI BRD score = 0) and head shake or ear flick (WI BRD score = 1) and a second level for a unilateral or bilateral ear droop, or head tilt (WI BRD score = 2 or 3). All coefficients in this model were significant. Addition of the variables depression ($\Delta {\mathrm{G}}_{\mathrm{Depression}}^{2}=0.16$, *p* = 0.69), sex ($\Delta {\mathrm{G}}_{\mathrm{sex}}^{2}=1.5$, *p* = 0.22), or FPT ($\Delta {\mathrm{G}}_{\mathrm{FPT}}^{2}=0.0$, *p* = 0.98) did not significantly improve model fit or substantially change the values of other coefficients when entered into the model. The estimated coefficients of the BRD1 model are summarized in Table 2.

**Table 2:** Summary of conditional logistic regression model BRD1 parameters, including estimated parameter value (β_*p*_), estimated parameter standard error (S.E. (β_*p*_)), standardized *Z*-score (*Z*) and the 2-sided significance of the *Z*-score (*p*) and weighting score factors for the BRD1 clinical scoring system (*S_p_*) developed from the model based on 809 pairs of Holstein calves (1618 calves in total) prior to weaning and housed on a calf ranch in California’s San Joaquin Valley.

Clinical sign	Level	β_*p*_	S.E. (β_*p*_)	*Z*	*p*	*S_p_*
Cough	None	Referent				0
	Any	2.237	0.602	3.71	<0.0005	2
Nasal discharge	None	Referent				0
	Any	3.459	0.757	4.57	<0.0005	3
Ocular discharge	None	Referent				0
	Any	1.534	0.687	2.23	0.026	2
Ear position	Normal, ear flick or head shake	Referent				0
	Ear droop or head tilt	4.563	1.510	3.02	0.002	5
Rectal temp	<39.2°C	Referent				0
	≥39.2°C	1.552	0.626	2.48	0.013	2
Abnormal respiration	Absent	Referent				0
	Present	1.732	0.883	1.96	0.050	2

#### BRD2

The second selection process started with a model that included all levels of severity for the nasal discharge, ocular discharge, and head and ear position clinical signs as described in the WI BRD score and rectal temperature dichotomized at 39.2°C. The variable cough was dichotomized with the referent level including no cough (WI BRD score = 0) or any induced cough (WI BRD score = 1 or part of 2), contrasted to the second level including occasional or repeated spontaneous cough (WI score part of 2 or 3).

The best fit model that resulted from the selection process included the variables for ocular discharge, ear and head carriage, abnormal respiratory, and temperature dichotomized as in BRD1, the variable for cough dichotomized as described for BRD2, and nasal discharge categorized into three levels of severity: normal/no discharge (WI BRD score = 0) as the referent level versus mild, unilateral, and watery discharge (WI BRD score = 1) versus moderate or severe nasal discharge (moderate, copious, mucoid, purulent, bilateral, WI BRD score = 2 or 3). Model fit was not significantly improved when depression ($\Delta {\mathrm{G}}_{\mathrm{Depression}}^{2}=0.34$, *p* = 0.56), sex ($\Delta {\mathrm{G}}_{\mathrm{sex}}^{2}=1.65$, *p* = 0.20), or FPT ($\Delta {\mathrm{G}}_{\mathrm{FPT}}^{2}=0.1$, *p* = 0.80) were included in the model, nor did their inclusion substantially change the values of other coefficients when entered into the model. The second selected model and its coefficients are summarized in Table 3. All coefficient estimates in the model were significant except for the estimated coefficient for ocular discharge (*p* = 0.69). However, removal of the ocular discharge term caused a significant change in model fit (ΔG^2^ = 3.91, *p* = 0.048) and was therefore retained in the final model.

**Table 3:** Summary of conditional logistic regression model BRD2 parameters, including estimated parameter value (β_*p*_), estimated parameter standard error (S.E. (β_*p*_)), standardized *Z*-score (*Z*) and the 2-sided significance of the *Z*-score (*p*) and weighting score factors for the BRD2 clinical scoring system (*S_p_*) developed from the model based on 809 pairs of Holstein calves (1618 calves in total) prior to weaning and housed on a calf ranch in California’s San Joaquin Valley.

Clinical sign	Level	β_*p*_	S.E. (β_*p*_)	*Z*	*p*	*S_p_*
Cough	None or induced cough	Referent				0
	Spontaneous cough	2.150	0.854	2.52	0.012	2
Nasal discharge	None	Referent				0
	Mild, watery, unilateral	3.229	0.956	3.38	0.001	3
	Moderate or severe, mucoid or mucopurlent, bilateral	5.005	1.273	3.93	<0.0005	5
Ocular discharge	None	Referent				0
	Any	1.368	0.753	1.82	0.069	1
Ear position	Normal, ear flick or head shake	Referent category				0
	Ear droop or head tilt	5.213	2.134	2.44	0.015	5
Rectal temp	<39.2°C	Referent				0
	≥39.2°C	1.962	0.593	3.31	0.001	2
Abnormal respiration	Absent	Referent				0
	Present	1.834	0.838	2.19	0.029	2

#### BRD3

A third model was fit with only dichotomized predictors (as in BRD1) and that did not require laryngeal manipulation to induce a cough (as in BRD2). The third model was fit using the following dichotomized variables: nasal discharge dichotomized with normal signs (WI score 0) as the referent level versus any abnormal discharge (WI score 1, 2, or 3), ocular discharge dichotomized with no discharge (WI score 0) as the referent level versus any abnormal discharge (WI score 1, 2, or 3), cough dichotomized with no spontaneous cough (WI score 0, 1, or part of 2) as the referent level versus any spontaneous cough (WI score part of 2 or 3), ear and head position dichotomized with no ear droop, head tilt, or ear flick (WI score 0 or 1) as the referent level versus any ear droop or head tilt (WI score 2 or 3), rectal temperature dichotomized as described above, and abnormal respiration with eupneic as the referent level versus dyspneic, tachypneic or both. Model fit was not significantly improved when depression ($\Delta {\mathrm{G}}_{\mathrm{Depression}}^{2}=1.17$, *p* = 0.28), sex ($\Delta {\mathrm{G}}_{\mathrm{sex}}^{2}=2.17$, *p* = 0.14), or FPT ($\Delta {\mathrm{G}}_{\mathrm{FPT}}^{2}=0.05$, *p* = 0.83) were added to the model, nor did inclusion substantially change the values of other coefficients in the model. This final model was fit to create a system that included only dichotomous clinical signs and minimized calf handling in terms of laryngeal manipulation, which can be time consuming and a biosecurity concern because it often required entry into the hutch. The BRD3 model coefficients are summarized in Table 4.

**Table 4:** Summary of conditional logistic regression model BRD3 parameters, including estimated parameter value (β_*p*_), estimated parameter standard error (S.E. (β_*p*_)), standardized *Z*-score (*Z*) and the 2-sided significance of the *Z*-score (*p*) and weighting score factors for the BRD3 clinical scoring system (*S_p_*) developed from the model based on 809 pairs of Holstein calves (1618 calves in total) prior to weaning and housed on a calf ranch in California’s San Joaquin Valley.

Clinical sign	Level	β_*p*_	S.E. (β_*p*_)	*Z*	*p*	*S_p_*
Cough	None or induced cough	Referent				0
	Spontaneous cough	2.345	0.855	2.74	0.006	2
Nasal discharge	None	Referent				0
	Any	3.937	0.884	4.45	<0.0005	4
Ocular discharge	None	Referent				0
	Any	1.934	0.725	2.67	0.008	2
Ear position	Normal, ear flick or head shake	Referent				0
	Ear droop or head tilt	4.816	1.625	2.96	0.003	5
Rectal temp	<39.2°C	Referent				0
	≥39.2°C	1.902	0.562	3.38	0.001	2
Abnormal respiration	Absent	Referent				0
	Present	2.015	0.837	2.41	0.016	2

The three model-based scoring systems had similar performances classifying calves as BRD-positive or negative. The BRD1 system provided the best fit to the data (AIC_BRD1_ = 62.30) as it was developed using only data-driven methods. The BRD2 model included the variable cough specified to contrast any frequency of spontaneous cough (single or repeated) against the referent level, which included no cough or any induced cough, and thereby removing laryngeal palpation from the system. However, the best fit model for BRD2 required two discrete levels of abnormal nasal discharge and produced a model with a higher AIC estimate than that for BRD1 (AIC_BRD2_ = 68.76). The BRD3 model dichotomized the cough variable to eliminate laryngeal palpation from the system as was done in the BRD2 model and dichotomized the nasal discharge variable for simplicity as was done in BRD1. The final BRD3 model with the variables forced resulted in a slightly higher AIC value compared to BRD1, and a fit similar to BRD2 models (AIC_BRD3_ = 69.66).

### Scoring systems

The *S_p_* values of the BRD1 scoring system ranged from 2 to 5, and calves total scores ranged from 0 to 16. The median BRD1 score for cases was 9, and 90% of cases had a score of 5 or higher. The median BRD1 score for controls was 0, and 90% of controls had a score of 4 or less.

The *S_p_* values of the BRD2 scoring system ranged from 1 to 5, and individual total scores ranged from 0 to 17. The median BRD2 score for cases was 9, and 90% of cases had a score of 4 or higher. The median BRD2 score for controls was 0, and 90% of controls had a score of 4 or less.

The *S_p_* values of the BRD3 scoring system ranged from 2 to 5, and individual total scores ranged from 0 to 17. The median BRD3 score for cases was 8, and 90% of cases had a score of 4 or higher. The median BRD3 score for controls was 0, and 90% of controls had a score of 4 or lower. The frequency and *S_p_* values associated with clinical signs are summarized in Table 5.

**Table 5:** Score weights assigned to and frequency of respiratory clinical signs for 3 clinical scores (BRD1, BRD2, BRD3) from a sample of 2030 Holstein bull and heifer calves prior to weaning and housed on a calf ranch in California’s San Joaquin Valley. The diagnostic cut points for BRD1, BRD2, and BRD3 were 4, 4, and 5, respectively.

Clinical sign	Level	*S_p_* ^a^			Frequency	
		BRD1	BRD2	BRD3	Case	Control
Nasal discharge	Normal serous discharge	0	0	0	240	981
	Small amount of unilateral cloudy discharge	3	3	4	239	132
	Bilateral, cloudy, or excessive mucus discharge	3	5	4	322	39
	Copious bilateral mucopurulent discharge	3	5	4	68	9
Ocular discharge	Normal	0	0	0	586	1058
	Small amount of ocular discharge	2	1	2	182	80
	Moderate amount of bilateral discharge	2	1	2	87	21
	Heavy ocular discharge	2	1	2	14	2
Rectal temperature	<100.9	0	0	0	28	260
	101.0–101.9	0	0	0	112	630
	102.0–102.4	0	0	0	126	173
	102.5–102.9	2	2	2	128	52
	=>103.0	2	2	2	475	46
Ears & Head	Normal	0	0	0	387	1104
	Ear flick or head shake	0	0	0	181	25
	Slight unilateral droop	5	5	5	200	21
	Head tilt or bilateral droop	5	5	5	101	11
Cough	None	0	0	0	236	1054
	Single induced	2	0	0	99	34
	Repeated induced	2	0	0	161	24
	Occasional spontaneous	2	2	2	242	34
	Repeated Spontaneous	2	2	2	131	15
Abnormal respiration	Negative	0	0	0	402	1109
	Positive	2	2	2	467	52

**Notes.**

a Zeroes indicate referent levels.

### Determination of optimal cut-points

The proportion of cases, controls, and all enrolled calves correctly classified and the likelihood ratio positive (LR+) at each possible *S_cp_* for the BRD1, BRD2, and BRD3 systems are summarized in Tables 6, 7 and 8 respectively. The *S*_*cp*,optimal_ value for BRD1 was 4, which correctly classified 95.4% of the cases, 88.6% of the controls, and 91.5% of all of the calves in the study. The *S*_*cp*,optimal_ value for BRD2 was 4, which correctly classified 92.8% of the cases, 89.3% of the controls, and 90.8% of all of the calves in the study. The *S*_*cp*,optimal_ value for BRD3 was 5, which correctly classified 89.4% of the cases, 90.8% of the controls, and 90.2% of all of the calves in the study. The performance of each of the BRD systems at their respective *S*_*cp*,optimal_ and their agreement with the WI system are summarized in Table 9.

**Table 6:** Diagnostic performance for the BRD1 scoring system to correctly identify calves with bovine respiratory disease (BRD), calves without BRD (controls), all calves, and likelihood ratio positive (LR+) in a sample of 2030 Holstein bull and heifer calves prior to weaning and housed on a calf ranch in California’s San Joaquin Valley. All discrete values of *S_cp_* are presented except, 0, which was non-informative. The decision rule used to classify calves as score positive for BRD if *S*_total_ ≥ *S_cp_*, and score negative otherwise. The greatest proportion of calves identified over all of the possible cut-points was 91.5% when the cut-point was set to 4.

*S_cp_*	Pr (*S*_total_ > = *S_cp_*|Case)	Pr (*S*_total_ < *S_cp_*|Control)	Total % correctly classified	LR+^a^
2	96.2%	74.3%	83.7%	4
3	95.9%	81.4%	87.6%	5
4	95.4%	88.6%	91.5%	8
5	91.1%	90.0%	90.5%	9
6	85.9%	92.4%	89.6%	11
7	78.8%	93.3%	87.1%	12
8	59.3%	95.4%	80.0%	13
9	56.4%	95.5%	78.8%	13
10	32.7%	97.2%	69.6%	12
11	26.4%	98.0%	67.3%	13
12	16.9%	98.7%	63.7%	13
13	7.9%	99.2%	60.2%	10
14	7.7%	99.3%	60.1%	11
16	0.4%	99.9%	57.3%	4

**Notes.**

a Positive likelihood test ratio is the probability of a positive test result (*S*_total,*i*_ ≥ *S_cp_*) in a calf that has BRD divided by the probability of a positive test result in a calf without BRD.

**Table 7:** Diagnostic performance for the BRD2 scoring system to correctly identify calves with bovine respiratory disease (BRD), calves without BRD (controls), all calves, and likelihood ratio positive (LR+) in a sample of 2030 Holstein bull and heifer calves prior to weaning and housed on a calf ranch in California’s San Joaquin Valley. All discrete values of *S_cp_* are presented except, 0, which was non-informative. The decision rule used was to classify calves as score positive for BRD if *S*_total_ ≥ *S_cp_*, and score negative otherwise. The greatest proportion of calves identified over all of the possible cut-points was 90.8% when the cut-point was set to 4 (*S*_*cp* optimal_ = 4).

*S_cp_*	Pr (*S*_total_ > = *S_cp_*|Case)	Pr (*S*_total_ < *S_cp_*|Control)	Total % correctly classified	LR+^a^
1	96.1%	75.5%	84.3%	4
2	95.9%	79.7%	86.6%	5
3	94.6%	81.7%	87.2%	5
4	92.8%	89.3%	90.8%	9
5	88.8%	91.0%	90.1%	10
6	84.1%	92.7%	89.0%	11
7	74.6%	93.7%	85.5%	12
8	61.3%	95.1%	80.6%	12
9	51.7%	96.2%	77.1%	14
10	37.2%	97.3%	71.6%	14
11	26.0%	98.7%	67.6%	20
12	18.9%	99.1%	64.7%	20
13	10.1%	99.4%	61.2%	17
14	8.1%	99.4%	60.3%	13
15	3.0%	99.6%	58.2%	7
16	1.6%	99.8%	57.8%	9
17	0.1%	0.0%	57.2%	0

**Notes.**

a Positive likelihood test ratio is the probability of a positive test result (*S*_total,*i*_ ≥ *S_cp_*) in a calf that has BRD divided by the probability of a positive test result in a calf without BRD.

**Table 8:** Diagnostic performance for the BRD3 scoring system to correctly identify calves with bovine respiratory disease (BRD), calves without BRD (controls), all calves, and likelihood ratio positive (LR+) in a sample of 2030 Holstein bull and heifer calves prior to weaning and housed on a calf ranch in California’s San Joaquin Valley. All discrete values of *S_cp_* are presented except, 0, which was non-informative. The decision rule used was to classify calves as score positive for BRD if *S*_total_ ≥ *S_cp_*, and score negative otherwise. The greatest proportion of calves identified over all of the possible cut-points was 90.2% when the cut-point was set to 5 (*S*_*cp* optimal_ = 5).

*S_cp_*	Pr (*S*_total_ > = *S_cp_*|Case)	Pr (*S*_total_ < *S_cp_*|Control)	Total % correctly classified	LR+^a^
2	96.1%	75.5%	84.3%	4
4	94.6%	81.7%	87.2%	5
5	89.4%	90.8%	90.2%	10
6	89.1%	90.9%	90.1%	10
7	72.3%	93.7%	84.5%	11
8	69.5%	94.1%	83.6%	12
9	48.5%	96.6%	76.0%	14
10	42.1%	97.1%	73.6%	14
11	27.4%	97.9%	67.7%	13
12	15.9%	98.9%	63.4%	14
13	13.7%	99.2%	62.6%	18
15	5.2%	99.4%	59.1%	9
17	0.2%	99.9%	57.2%	3

**Notes.**

a Positive likelihood test ratio is the probability of a positive test result (*S*_total,*i*_ ≥ *S_cp_*) in a calf that has BRD divided by the probability of a positive test result in a calf without BRD.

**Table 9:** The optimal cut-points, summary of diagnostic performance at their respective optimal cut-points, and Cohen’s kappa values with the WI score for BRD1, BRD2, and BRD3 based on sample 2030 Holstein bull and heifer calves prior to weaning and housed on a calf ranch in California’s San Joaquin Valley.

	BRD1	BRD2	BRD3
*S*_*cp*,optimal_	4	4	5
Total % correctly identified	91.5%	90.8%	90.2%
Pr(*S*_total_ ≥ *S*_*cp*,optimal_|Case)	95.4%	92.8%	89.4%
Pr(*S*_total_ < *S*_*cp*,optimal_|Control)	88.6%	89.3%	90.8%
Kappa	0.959	0.944	0.916

### Agreement of tests

Cohen’s kappa values for agreement between the WI score and BRD1, BRD2, and BRD3 were 0.96, 0.94, and 0.92, respectively. These kappa values were all greater than 0.75, which indicated excellent agreement beyond chance between the WI score results and the results of each of the three BRD scores (Fleiss, Levin & Paik, 2003).

## Discussion

Three scoring systems were proposed for on-farm use to diagnose BRD in pre-weaned dairy calves. The score weights and cut-points of these scoring systems were selected using statistical estimation, in contrast to previously described systems in which score weights and cut-points were determined subjectively. All three scoring systems had excellent agreement with the WI score. The BRD1 scoring system correctly classified 91.5% of the calves in the study, but required handling calves without spontaneous cough in an attempt to elicit an induced cough. The BRD2 scoring system correctly classified 90.8% of the calves in the study and did not require additional calf handling to induce coughing, but had three levels of nasal discharge, instead of two. The BRD3 scoring system correctly classified 90.2% of the enrolled calves, did not require additional calf handling to attempt inducing a cough, and included only dichotomous predictors for all the clinical signs. Given similar performance to the WI scoring systems, our BRD scoring systems require less qualitative assessment of clinical signs and hence offer simpler algorithms for on-farm diagnosis of BRD in pre-weaned dairy calves. Specifically, BRD3 would be most feasible on dairies and calf raising facilities for daily observation of large calf numbers in intensive dairy systems, this is due to the simplicity of the dichotomized clinical signs and reduced calf handling required to obtain the final score. Specifically, the BRD3 scoring system will require handling a calf for rectal temperature measurement only when a calf’s total score based on all other clinical signs is equal to or greater than 4. At that time, a rectal temperature of 39.2°C (102.5°F) or greater will increase the calf’s score beyond the scoring system’s cut-point of 5. Nevertheless, the three scoring systems developed in this study are described and presented to demonstrate the selection process and allow end users to select the system that best suits their needs.

The systems presented here are the first clinical scoring systems for BRD published in peer-reviewed literature for which clinical data was used to weight scores and set cut-points. Prior to this study, the WI scoring system was the most widely accepted scoring system used in dairy medicine, but important pieces of information about this system, such as methods used to assign *S_cp_* and *S_p_*, sensitivity, specificity, predictive values and reliability, are absent from peer-reviewed literature. Further, the WI system uses 5 clinical predictors, each with 4 levels, and only uses four of these predictors to assign a score to the calf since the lower score of eyes and ears are dropped from the score. The systems presented here use 6 clinical predictors each with 2 levels, with the exception of nasal discharge in the BRD2 score, which has 3 levels, and use all 6 clinical predictors to assign total scores. It is anticipated that the inclusion of fewer levels of clinical predictors will improve the reliability and acceptance of the score.

The task of accurately classifying calves as BRD-positive cases or BRD-negative controls is difficult without a reference test. Identification of cases and controls without a gold-standard is a common challenge in epidemiologic studies. Case definitions that are based on multiple criteria are employed frequently and considered suitable so long as the criteria are appropriate for the goals of the study (Coggon et al., 2005). The algorithm, shown in Fig. 1, was developed to best identify cases and controls based on the available data. The detection of a primary pathogen for BRD, such as BRSV, using a sensitive and specific method, such as qPCR, indicates a high index of suspicion that the pathogen is present and the calf has BRD. The isolation of opportunistically pathogenic organisms, such as *P. multocida* and *Mycoplasma bovis*, is a much less specific criterion for BRD because these bacteria are commonly isolated from cattle without BRD. The specificity of bacterial isolation was improved by incorporating clinical information quantified by the WI scores. The WI system can be a useful method to quantify clinical BRD signs and was effectively employed in this study because two of the researchers were veterinarians with substantial calf experience which allowed for consistent evaluation and scoring of calves for BRD at enrollment. Classification as a case using the combined results of the WI score and bacterial isolation is a form of serial test interpretation, a strategy used to improve test specificity and reduce false positive results (Thurmond & Johnson, 2004).

As diagnostic tools, BRD scoring systems are most informative when estimates of the test sensitivity and specificity are known (Dohoo, Martin & Stryhn, 2010). The conditional probability that *S*_*i*,total_ ≥ *S_cp_* given *i* was a case, and that *S*_*i*,total_ < *S_cp_* given that *i* was a control, could be interpreted as cut-point specific estimates of sensitivity and specificity, respectively. However, these values were not interpreted as sensitivity and specificity in this study because of concerns that these estimates may be biased compared to the true sensitivity and specificity, due to two potential sources of selection bias. The first source arises from the use of the WI score in the case definition because the clinical signs used by the WI score were also included as predictors in the models for the BRD1, BRD2, and BRD3 systems. Hence, sampling of the cases and controls may not have been independent of the predictors in the model, resulting in biased parameter estimates. Although it is expected that the incorporation of microbiological results would reduce such a bias, caution is required when interpreting the estimated coefficients as causal measures of association. However, since the estimated coefficients were used to assign relative score weights, not to quantify causal associations, the method is acceptable for this application. The second source of bias arises from the pair-matched design of original study, which may have caused the sampled controls to be more similar to the cases and not representative of the referent population of all healthy calves on the facility. The CLR models account for the artificial similarities between cases and controls, but the ROC analysis used to select the optimal cut-points does not. Further research is needed to estimate the sensitivity and specificity of all three presented BRD scoring systems. Diagnostic methods such as thoracic ultrasound, auscultation, and hematology should be used to diagnose BRD in calves and estimate the sensitivity and specificity. While the methods listed are not gold-standards for BRD diagnosis, methods of estimating test sensitivity and specificity using imperfect tests have been described (Enøe, Georgiadis & Johnson, 2000). Furthermore, any proposed scoring systems should be validated using an independent data set.

Bovine coronavirus was not included in the case assignment algorithm due to the PCR lack of specificity for the respiratory BCoV subtype and the unclear role of BCoV in BRD in calves. While multiple subtypes of BCoV in cattle have been described, including respiratory and enteric calf diarrhea subtypes, based on the clinical presentation of infected cattle, no antigenic or genetic markers have been found to consistently differentiate the subtypes (Saif, 2010). Previous research has also established that animals infected with either subtype will shed viral particles in nasal secretions (Cho et al., 2001a; Cho et al., 2001b; Hasoksuz et al., 2002). Hence, the results of the BCoV PCR cannot be relied on to distinguish respiratory BCoV from enteric strains being shed from nasal mucosa. Inclusion of the BCoV term as a predictor did not significantly improve the fit of the model ($\Delta {\mathrm{G}}_{\mathrm{BRD}1}^{2}=0.51$, *p* = 0.439; $\Delta {\mathrm{G}}_{\mathrm{BRD}2}^{2}=0.10$, *p* = 0.75; $\Delta {\mathrm{G}}_{\mathrm{BRD}3}^{2}=0.10$, *p* = 0.75) and did not meaningfully change the values of the other coefficients. Additionally, the role of BCoV in calf BRD is unclear. Experimentally, BCoV has been demonstrated to induce respiratory signs following oronasal inoculation in calves in some studies (Kapil et al., 1991; McNulty et al., 1984) and not in others (Reynolds et al., 1985; Saif et al., 1986). Similarly, some observational studies have found significant associations between BCoV exposure and BRD (Storz et al., 2000), while others were unable to detect an association between serologic evidence of exposure to BCoV and incidence of BRD (Martin et al., 1998; Plummer et al., 2004).

While BHV-1, BVD, and BRSV are all BRD pathogens, no calves from the study tested positive for BHV-1 or BVDV. Several potential explanations exist for why these viruses were not detected. A greater proportion of cattle in the western US are vaccinated for BHV-1 and BVDV than other parts of the United States (USDA, 2007). It has been shown that increasing the proportion of vaccinated individuals causes an increase in age at infection thereby lowering disease prevalence in younger populations (Keeling & Rohani, 2008). A 2003 study of dairy calves in this region of California found only two calves out of 434 (0.5%) from two herds to be persistently infected (PI) with BVDV at birth (Munoz-Zanzi et al., 2003). It is expected that the prevalence of persistently infected calves on this facility in 2011 was even lower due to the facility’s selectivity of clients and the increased mortality of PI calves during the first few weeks of life. Since PCR assays for each calf were performed using material collected and stored in a single vial, the presence of positive results for BRSV and BCoV (both enveloped RNA viruses) would seem to indicate sample handling was also sufficient for BHV-1 and BVDV (enveloped DNA and RNA viruses, respectively) had they been present. The absence of BHV-1 and BVDV in this population, and subsequent omission of these viruses from the case definition algorithm, may limit the validity of the presented systems to detect cases of BRD in other populations where these pathogens may be more prevalent. Further studies in calves infected with BHV-1 and BVD viruses are required to validate the accuracy of the current scoring systems.

The current study was performed in the United States, where rectal temperature is typically measured in degrees Fahrenheit. While rectal temperature was dichotomized using the reported upper physiological limit in dairy cattle (39.2°C or 102.5°F) (Andersson & Jonasson, 1993), other temperature cut-points were also evaluated, including less than, or greater than to or equal to 38.3°C (101.0°F), 38.6°C (101.5°F), 38.9°C (102.0°F), and 39.4°C (103.0°F). However, none of the models with these alternate temperature cut-points fit models as well as 39.2°C or 102.5°F (data not shown).

The values of *S_p_* in this study were assigned based on the values of the CLR coefficients. This approach to *S_p_* values assignment was adapted from a method described previously (Segev et al., 2008), which assigned *S_p_* values based on odds ratios (ORs) estimated by exponentiating logistic regression coefficients. This change was made because logistic regression models assume that the joint effects of variables relate to the odds of an outcome in a multiplicative, not additive, manner (Breslow & Day, 1980). Therefore, the sum of the ORs as determined by multivariable logistic regression does not necessarily represent the OR of disease in an individual with multiple exposures compared to an individual with no exposures. Another method that determines *S_p_* values based on logistic regression coefficients instead of ORs has been described (Sullivan, Massaro & D’Agostino, 2004); however the pair-matched case-control design of the current study was incompatible with Sullivan’s method.

## Conclusion

The BRD scoring systems developed in this study provide three options to assess the BRD status of pre-weaned dairy calves. The scoring systems utilize objective and easy to obtain criteria thereby reducing subjectivity. For ease of use, an investigator may assess the presence or absence of ocular discharge, nasal discharge, ear droop or head tilt, respiratory quality and spontaneous coughing. Calves with abnormal ear or head carriage, or calves with nasal discharge and one other clinical sign, or calves that have any three clinical signs are BRD cases based on the BRD3 scoring system. Only calves with nasal discharge or calves with two other clinical signs (spontaneous coughing, ocular discharge or abnormal respiratory) would require handling the calf to measure its rectal temperature and confirm BRD status if the temperature is ≥39.2°C or 102.5°F.
